# Isolated right superior vena cava draining into the left atrium in a child with vein of Galen aneurysmal malformation—case report

**DOI:** 10.1186/s13019-018-0758-x

**Published:** 2018-06-13

**Authors:** Ahmed F. Elmahrouk, Abdelmonem Helal, Mohamed F. Ismail, Tamer Hamouda, Mohammed Mashali, Ahmed A. Jamjoom, Jameel A. Al-Ata

**Affiliations:** 1 Cardiothoracic Surgery Department, King Faisal Specialist Hospital and Research Center, Jeddah, Saudi Arabia; 2 Department of Pediatric Cardiology, King Faisal Specialist Hospital and Research Center, Jeddah, Saudi Arabia; 3 Cardiothoracic Surgery Department, Faculty of Medicine, Tanta University, Tanta, Egypt; 4 Department of Pediatric Cardiology, Qasr Alainy Faculty of Medicine, Cairo University, Cairo, Egypt; 5 Cardiothoracic Surgery Department, Faculty of Medicine, Mansoura University, Manasoura, Egypt; 6 Cardiothoracic Surgery Department, Faculty of Medicine, Benha University, Benha, Egypt; 7 Department Of Pediatrics, Faculty of Medicine, King Abdulaziz University, Jeddah, Saudi Arabia

**Keywords:** Vein of Galen aneurysmal malformations, Partial anomalous systemic venous drainage, Endovascular embolization

## Abstract

**Background:**

Isolated right Superior Vena Cava drainage into the left atrium in the absence of other cardiac anomalies is an extremely rare condition. The vein of Galen aneurysmal malformation is a congenital vascular malformation. It comprises 1% of all pediatric congenital anomalies. The association vein of Galen aneurysmal malformation, with congenital heart disease has been described.

**Case presentation:**

We describe a 16-months old toddler presenting at 7-months of age with respiratory distress and cyanosis. CT brain showed Vein of Galen aneurysmal malformations. Echocardiography showed partial anomalous systemic venous drainage in the form of right superior vena cava drained into left atrium. Four sessions of Endovascular embolization were performed. Surgical repair of partial anomalous systemic venous drainage was done successfully.

**Conclusions:**

The superior vena cava in our case overrides the atrial septum promoting direct drainage of venous return into the LA, thus causing dilated left ventricle instead of dilatation of right ventricle which is the usual presentation of VAGMs.

## Background

Isolated right Superior Vena Cava (RSVC) drainage into the left atrium (LA) in the absence of other cardiac anomalies is an extremely rare condition. It accounts for approximately 0.5% of congenital cardiac cases [1]. The vein of Galen aneurysmal malformation (VGAM) is a congenital vascular malformation. It manifests in the first year of birth with high output cardiac failure, and comprises 30% of the pediatric vascular and 1% of all pediatric congenital anomalies [2].

## Case presentation

A sixteen-months toddler, initially diagnosed at the age of 7 months, to have VGAM and anomalous systemic venous drainage of the RSVC to LA. He first came to the emergency department with respiratory distress, mild cyanosis and moderate hydrocephalus.

**Fig. 1:**
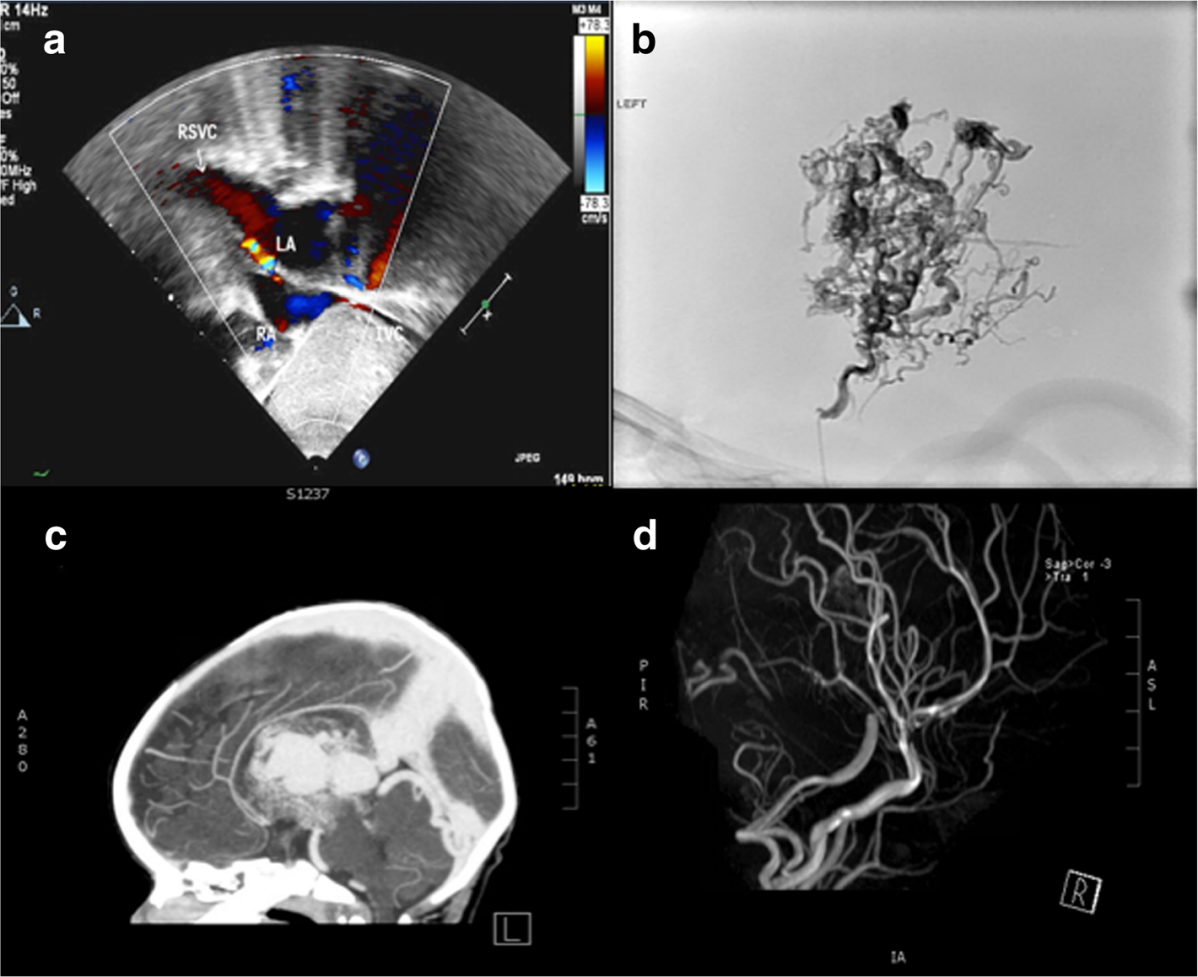
**a** Echocardiography modified bicaval view showing dilated right superior vena cava (RSVC) draining into dilated the dilated left atrium (LA). Inferior vena cava (IVC) is draining normally to the right atrium (RA). **b** Selective Angiography with multiple supplying vessels of a large arteriovenous malformation deeply located in the left basal ganglia with drainage into MProsV of Markowski that drains into the vein of Galen. **c** Moderate hydrocephalus is present. The maximum size of the arteriovenous malformation is approximately 4.9 cm in size. The Arterial supply from middle cerebral artery and anterior cerebral artery small branches. The venous drainage through MProsV of Markowski that drains into the vein of Galen with significant dilatation of the torcula and the transverse sinuses. **d** MR angiography and MR venogram: Complete obliteration of the arteriovenous malformation and the arteriovenous shunting. No further embolization is needed

On arrival to the emergency room, he was alert, active, afebrile and hemodynamically stable. His oxygen saturation was 80% and head circumference was 45 cm. Chest-X-ray showed cardiomegaly and echocardiography revealed a dilated RSVC draining into the LA with a patent foramen oval shunting left to right. The pulmonary veins were well seen and were normally draining to the LA, whereas the inferior vena cava and the hepatic veins drain into the right atrium (RA). The LA and left ventricle were both mildly dilated (Fig. 1a).

A trans-fontanelle ultrasound showed dilated lateral ventricles and third ventricle with evidence of flow seen by color Doppler. Further evaluation by CT brain angiogram showed a large intraventricular hematoma in the left lateral ventricle, a 4.9 cm arteriovenous malformation with markedly distended venous arteries involving the basal ganglia extending to the left lateral ventricle (Fig. 1b).

The arterial supply was predominantly from middle cerebral artery and anterior cerebral artery small branches. The venous drainage was through the median prosencephalic vein (MProsV) of Markowski into the vein of Galen (Fig. 1c).

Four sessions of stepwise embolization of the VGAM was performed, with 2 months interval, however, he developed an intraventricular hemorrhage with progressive hydrocephalus and acute infarction in the left superior fronto-parietal region. Ventriculo-peritoneal shunt was inserted to decrease the intracranial pressure. After 4 months of follow up with pediatric neurology patient examination showed motor developmental delay in the form of delayed sitting without support and minimal right hemiparesis. Follow-up MRI brain showed evidence for left-sided subdural hygroma with associated minimal shift of the midline towards the right side and complete obliteration of the arteriovenous malformation and the arteriovenous shunting (Fig. 1d) The patient was cleared for cardiac procedure.

**Fig. 2:**
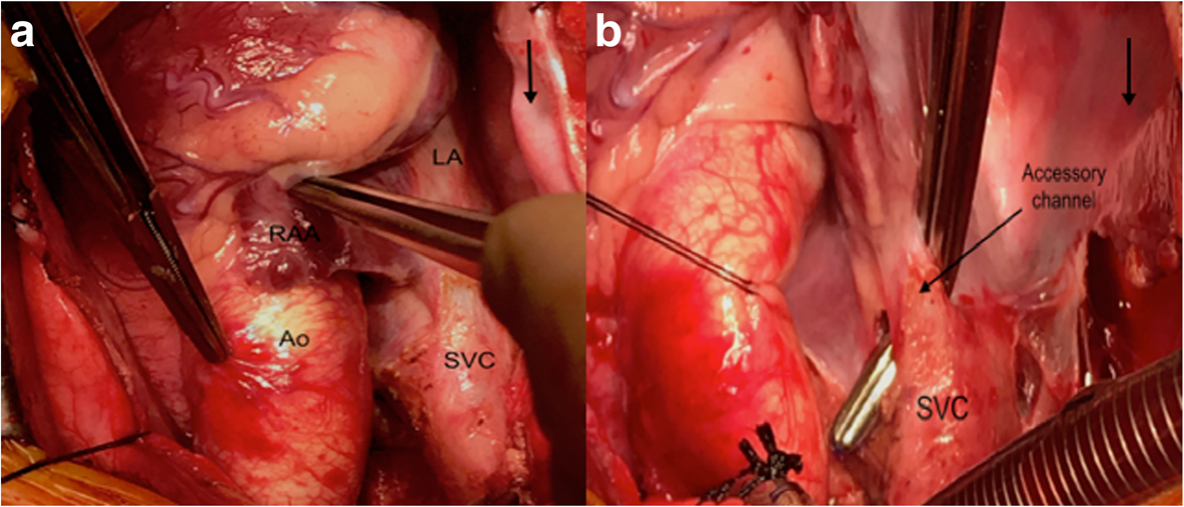
**a** Intra-operative view with Superior Vena Cava (SVC) draining into the Left Atrium (LA), The Aorta (Ao) is pulled with the left forceps and the Right Atrial appendage (RAA) is pulled with the right forceps. An arrow on the right angle (surgeon side) pointing toward the head of the patient. **b** Intra-operative view showing the right-angle forceps separating a small accessory channel originating from the SVC, the channel drains to the right atrium while the main SVC is draining to the left Atrium. An arrow on the right angle (surgeon side) pointing toward the head of the patient

**Fig. 3:**
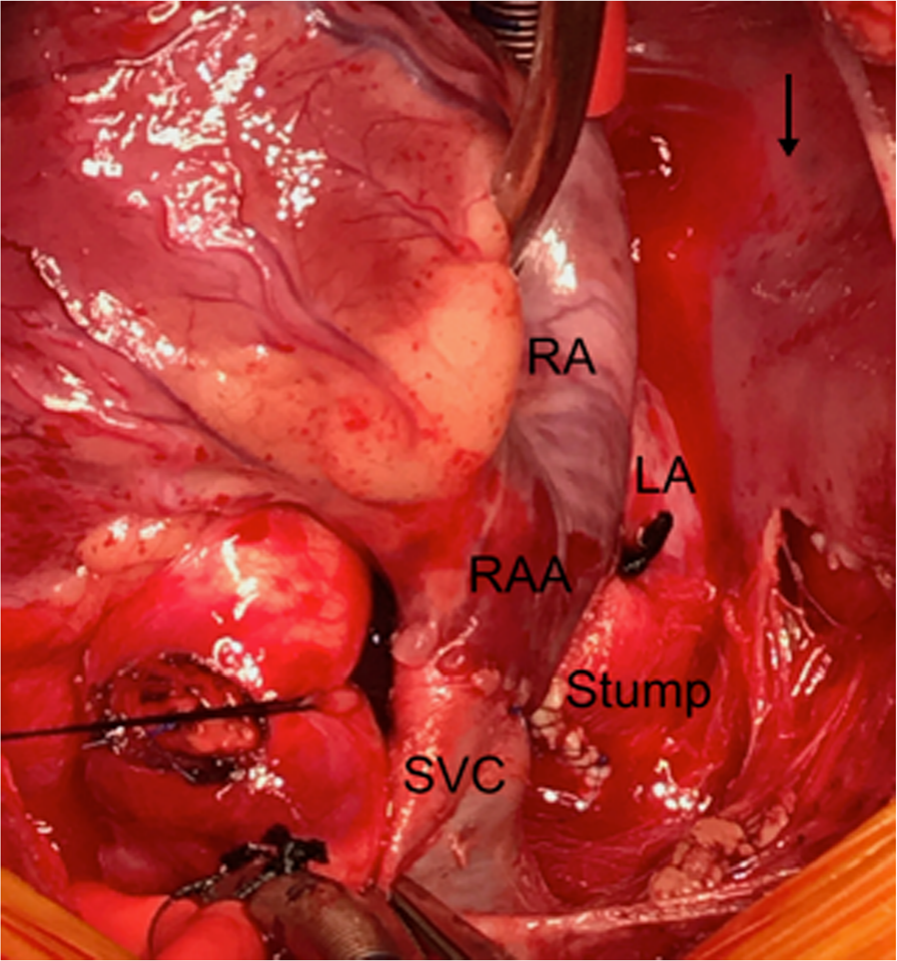
Intra-operative view showing the proximal SVC anastomosis with the RAA, and the distal SVC stump is over-sewn and the small channel is ligated. An arrow on the right angle (surgeon side) pointing toward the head of the patient

At the age of 16 months, the patient underwent surgical re-routing of the RSVC to the RA through median sternotomy using cardiopulmonary bypass. Intraoperative examination revealed posterior displacement of the RSVC which drain into the LA, there was no Left SVC (Fig. 2a) Just before the RSVC-LA connection a small separate channel was identified (Fig. 2b) The right atrial appendage was opened; this channel was found to drain into RA and no sinus venousus (SV) defect was found. The inferior vena cava and pulmonary veins drained normally. The RSVC was amputated 2 cm proximal to the RSVC-LA junction, the distal end of the RSVC was over-sewn and the proximal end was connected to the opening in the right atrial appendage (Fig. 3).

He was seen in the outpatient clinic with a remarkable improvement in cognitive and motor function with oxygen saturation above 97%.

## Discussion

The high-flow, low-resistance arteriovenous connection in VGAM causes a compensatory increase in blood volume and cardiac output and lead to High output cardiac failure [2]. in our case the presentation was different due to RSVC-LA, that leads to hemodynamic modification in the form of central cyanosis and increased left ventricular end diastolic pressure, and pulmonary venous congestion. It is important to recognize the potential importance of right-left shunt (RSVC-LA), which leads to a high volume of systemic venous return. Any connection between the venous and arterial systems poses a substantial risk of brain abscess and paradoxical embolization [2, 3].

The VGAM is the dominant lesion in neonates with associated congenital heart disease, its repair or palliation should take priority over correction of the cardiac defects. Endovascular intervention is the first-line of management [2, 3]. .Ideally, the treatment should start at 4–5 months of age to maximize efficacy of the intervention and minimize the risk of delay in cerebral maturation. Our patient was presented at the age of 7 months. A stepwise endovascular embolization is recommended to avoid rapid hemodynamic changes, which can lead to parenchymal hemorrhage or massive venous thrombosis [2].

The association between isolated RSVC drainage of into LA and VGAMs, can be explained by the increased venous return through the SVC in utero that could interfere with the absorption of the right horn of the SV. This may lead to a relative leftward and cephalic distortion of the right horn of the SV, this results in opening of the SVC into the LA [4, 5].

## Conclusion

In conclusion, the SVC in our case overrides the atrial septum promoting direct drainage of venous return into the LA, thus causing dilated left ventricle instead of dilatation of right ventricle which is the usual presentation of VAGMs.

## Abbreviations

(MProsV) of Markowski: The median prosencephalic vein
LA: Left Atrium
RA: The Right Atrium
RSVC: Right Superior Vena Cava
SV: Sinus Venousus
VGAM: The vein of Galen aneurysmal malformation

## References

[1] Baggett C, Skeen SJ, Gantt DS, Trotter BR, Birkemeier KL (2009). Isolated right superior vena cava drainage into the left atrium diagnosed noninvasively in the Peripartum period. Tex Heart Inst J.

[2] Recinos PF, Rahmathulla G, Pearl M, Recinos VR, Jallo GI (2012). Vein of Galen malformations: epidemiology, clinical presentations, management. Neurosurg Clin N Am.

[3] McElhinney DB, Halbach VV, Silverman NH, Dowd CF, Hanley FL (1998). Congenital cardiac anomalies with vein of Galen malformations in infants. Arch Dis Child.

[4] Kirsch WM, Carlsson E, Hartmann AF (1961). A case of anomalous drainage of the superior vena cava into the left atrium. J Thorac Cardiovasc Surg.

[5] Van Praagh S, Geva T, Lock JE, del Nido PJ, Vance MS, Van Praagh R. Biatrial or left atrial drainage of the right superior vena cava: anatomic, morphogenetic, and surgical considerations: report of three new cases and literature review. Pediatr Cardiol. 24:350–63. 10.1007/s00246-002-0329-7 12457258

